# Estimation of the time of death by measuring the variation of lateral cerebral ventricle volume and cerebrospinal fluid radiodensity using postmortem computed tomography

**DOI:** 10.1007/s00414-021-02698-6

**Published:** 2021-09-25

**Authors:** Fabio De-Giorgio, Gabriele Ciasca, Gennaro Fecondo, Alberto Mazzini, Marco De Spirito, Vincenzo L. Pascali

**Affiliations:** 1 Fondazione Policlinico Universitario A. Gemelli IRCCS, Rome, Italy; 2 Department of Healthcare Surveillance and Bioethics, Section of Legal Medicine, Università Cattolica del Sacro Cuore, Rome, Italy; 3 Neuroscience Department, Section of Physics, Università Cattolica del Sacro Cuore, Rome, Italy

**Keywords:** Postmortem changes, Postmortem interval, Time of death, Postmortem computed tomography, Lateral cerebral ventricle, Radiodensity, Cerebrospinal fluid

## Abstract

Using postmortem CT (PMCT), changes in the volume of the lateral cerebral ventricles (LCVs) and modifications of the radiodensity of cerebrospinal fluid (CSF) have been examined to identify a possible relationship between these changes and the time of death. Subsequent periodical CT scans termed “sequential scans” for ten corpses at known time of death were obtained, and a 3D segmentation of the entire LCV was carried out to measure its volume and radiodensity over time from ~ 5.5- h up to 273-h postmortem. A linear decrease of the LCV volume for all the cases was observed in the investigated time range, together with an overall logarithmic increase of radiodensity. Although a larger sampling should be performed to improve the result reliability, our finding suggests that the postmortem variation of CSF radiodensity can be a potentially useful tool in determining postmortem interval, a finding that is worthy of further investigation.

## Introduction

Although autopsies are still recognized as necessary and must be considered indefeasible, they are sometimes refused by the family or not tolerated for religious reasons. In these cases, a conventional autopsy could be replaced by some “alternative” procedures, such as postmortem imaging [1] or postmortem toxicology/biochemistry [2–4], though these are usually ancillary and supplemental to the classic postmortem investigation.

Postmortem imaging can be performed with the use of different technologies, such as computed tomography (CT) or magnetic resonance imaging (MRI). It can be used to complement traditional forensic autopsies; moreover, it is a non-invasive, repeatable technique [5] and grants forensic practitioners with unlimited access to the acquired data over time for post-autopsy analyses, and it is increasingly used as a tool for investigating causes and manner of death [1, 6].

Body changes that are observed after death play a crucial role when it comes to postmortem investigations; indeed, such alterations often mislead pathologists and hamper diagnoses. In this regard, it is worth mentioning the paper from Egger et al. [7], which derived a radiological alteration index (RAI) able to quantify postmortem body changes by measuring the presence of gas in the body using PMCT.

Postmortem body changes observed via PMCT can be also used to determine the postmortem interval (PMI) of a subject. Although several qualitative and quantitative approaches have been proposed in determining the time since death and the postmortem interval (PMI) [8–13], traditional methods are still predominantly used in forensic practice, and these methods are based on an evaluation of livor, rigor, and algor mortis.

Until now, only a few studies have been published on the utilization of PMCT as a tool for PMI determination, showing promising results. Despite this potential, the possibility to use PMCT alone, as the method of choice, is still being debated in the literature.

Hasegawa et al. observed a linear reduction with time in the size of the frontal and posterior horns of the lateral cerebral ventricles (LCV), as well as a progressive rise over time in the radiodensity of the cerebrospinal fluid (CSF) as a function of the PMI in three male subjects over time from 1–2-h up to 16–24-h postmortem (hpm) [11].

Koopmanschap et al. evaluated the postmortem changes in radiodensity of the CSF of three groups of subjects. The first scans of group A were taken over time from 6–10-h up to 36-h postmortem. The radiodensity values of the CSF were 5.7–8.1 HU at the first CT scans of the corpses and 10–12 HU at the last CT scans. In group B, the radiodensity of CSF was evaluated in 98 in-hospital corpses. The subjects underwent PMCT only once, with results ranging from 2 to 63.8 hpm. In group C – consisting of 12 subjects who died outside of the hospital – the CT scans were performed at a PMI of 15–42.3 hpm. In all three groups, the radiodensity of CSF increased linearly over time in all cases, showing a high correlation with the PMI. Groups B and C showed higher radiodensity values than group A. Groups B and C also showed a weaker relationship between the rise in the HU of the CSF and the PMI than did group A. In all the subjects, a linear relationship between a gradual increase in CSF radiodensity and the PMI was observed [14].

Morikawa et al. evaluated CSF density in 189 corpses. The corpses were divided into 3 groups based on the PMI (group A, postmortem days 0.5–2.5; group B, postmortem days 3–7; group C, postmortem days 10–30). The authors observed that the CSF radiodensity remained stable at a value of 20 HUs up until day 2.5, with few variations showing a statistically significant increasing pattern after day 3. The highest value was recorded on day 30. However, the increase was not linear with the PMI, and a discrete amount of overlap in terms of CSF radiodensity was observed [15].

In this study, we focus our attention on the postmortem changes in the volume of the LCVs, as well as the modifications of the radiodensity of CSF, to identify a possible relationship between these changes and the PMI.

## Materials and methods

### Subject recruitment and CT measurements

A total of 10 corpses were studied with a mean age at the time of death of 65.5 years (age range = 33–88 years; SD = 18.7) and an average BMI of 26.1 (SD = 3.5). Out of the 10 subjects, 5 were females and 5 were males (Table 1). Inclusion criteria were a defined time point of death that occurred during the day and with witnesses (as reported by medical doctors) and age above 18 years. Criteria used to exclude brains from the study were cerebral organic diseases and the presence of posttraumatic changes (i.e., head trauma following a car accident). Concerning cases E and F, which died as a consequence of a fall, cerebral organic diseases and/or posttraumatic changes were excluded based on the data from the first aid intervention, witnesses to the event, radiology (head CT), and autopsy (macroscopic and microscopic examination).

**Table 1:** Detailed list of the studied cases, provided with baseline parameters (gender, age), estimated time of death, cause of death, and the time interval between the first and the last PMCT scan

Case	Gender	Age	Cause of death	First PMCT scan (hours)	Last PMCT scan (hours)	Rectal temperature at first PMCT scan (°C)
A	F	78	Sudden cardiac death	7	20	31.2
B	M	83	Sudden cardiac death	26	50	18.0
C	M	53	Sudden cardiac death	5	94	32.8
D	M	84	Sudden cardiac death	8	11	30.3
E	F	45	Cardiac contusion	35	273	18.0
F	F	75	Lesion of the femoral artery	24	136	18.0
G	F	33	Ipovolemic shock	23	71	18.0
H	M	56	Sudden cardiac death	14	49	25.3
I	F	88	Sudden cardiac death	24	70	18.0
J	F	78	Sudden cardiac death	71	129	18.0

Each corpse underwent consecutive CT scans before autopsy to compare images of the same structures at different postmortem times. The exams were conducted with a Somatom Scope 16-slice CT scanner, Siemens Healthineers Italia. The CT scans were characterized by the following parameters: 130 kV, 150 mA, 2.4-mm slice thickness, using the H31S head-district kernel reconstruction. The corpses were placed on a horizontal CT table in a supine position with their arms at their sides. They were fully clothed and wrapped in body bags. Both cranial and full-body CT scans (from the skull vertex to the most distal point allowable, up to about 2000 mm) were obtained. No contrast agent was used in this procedure. Two sets of scans were performed: one from the skull vertex to the sternal notch (1-mm reconstructions) and one from the skull vertex to beyond the feet (1.5-mm reconstructions). Scans were evaluated by a radiologist and repeated if artifacts were present.

The corpses were kept in the same position for the entire procedure (from the first scan session to the last) to provide reliable and reproducible results. The temperature in the CT room was kept at 18 °C, and air humidity was set at 49%. To obtain core temperature values, rectal measurements were carried out for each case at the time of the first PMCT scan. The obtained temperature values were 18.0 °C (*N* = 6), 25.3 °C (*N* = 1), 30.3 °C (*N* = 1), 31.2 °C (*N* = 1), and 32.8 °C (*N* = 1). The corpses were then autopsied, and routine histological/toxicological analyses were performed. All conducted investigations, including the total-body CT examination and complete autopsy (macroscopic and microscopic examination, toxicological analyses), were authorized by the judicial authority.

### Image analysis

The PMCT images, imported in DICOM format, have been analyzed using ImageJ open-source software (1.53b 31 May 2020 Fiji release), together with the Segmentation Editor plugin available in the ImageJ online repository. To isolate the cerebral lateral ventricle region, the skull CT images have been suitably adjusted to obtain vertical alignment, equalized in terms of bit content in the range 0–255, and the first and the last percentiles were eliminated to stabilize the numerical values. Then, we segmented the slice regions corresponding to the LCV using the *segmentation editor* plugin, together with the *magic wand* tool, and then refining manually the results under the supervision of an expert pathologist. Volume estimation has been performed on the whole CT stack, multiplying the total area measured on all the selected slices by the distance between adjacent slices. For the radiodensity measures, instead, the software automatically returns the average and SD of HU values for each segmented CT slice, via the *Analyse*—> *Measure* menu. The mean radiodensity of the entire LCV was then estimated utilizing an average of the results obtained on each slice, weighted for the corresponding area. Finally, a 3D lateral ventricle reconstruction has been performed using the *ImageJ 3D viewer* plugin.

### Statistical methods

Statistical analyses were performed with the software package R (the R version 4.1.0 2021–05–18, was used for editing final figures). Data visualization was performed with the package *ggplot2*. Correlations among the different variables were evaluated using Spearman’s rank correlation coefficient, as described in [16]. Coefficients were arranged in a correlation map using the R package *corrplot*. The strength of the correlation was judged using coefficients of > 0.70 as strong, 0.30–0.70 as moderate, and < 0.3 as weak correlation. Calculations were performed with the function *rcorr* implemented in R. The same data were also fitted to a linear trend using the *lm* function. Non-linear fitting was performed with the *nls* function in R. Fitted curve were presented together with confidence bands, corresponding to one standard deviation, as described in [17]. Data measured in the present paper were systematically compared with those published in the pioneering work of Hasegawa and coworkers [11], which contributed to stimulate us to undertake this research and is published by the authors under the creative commons license (CC-BY 4.0). For a direct comparison among time trends, data points from [11] were digitalized from the published manuscript to obtain the *x* and *y* coordinates. The digitalization process might induce an interpolation error of the order of a tenth the size of the original dots. The overall contribution of this error has little effect on the fitted parameters (between two and three orders of magnitude less than the corresponding estimate). Additionally, volume values were converted from ml to mm^3^.

## Results

**Fig. 1:**
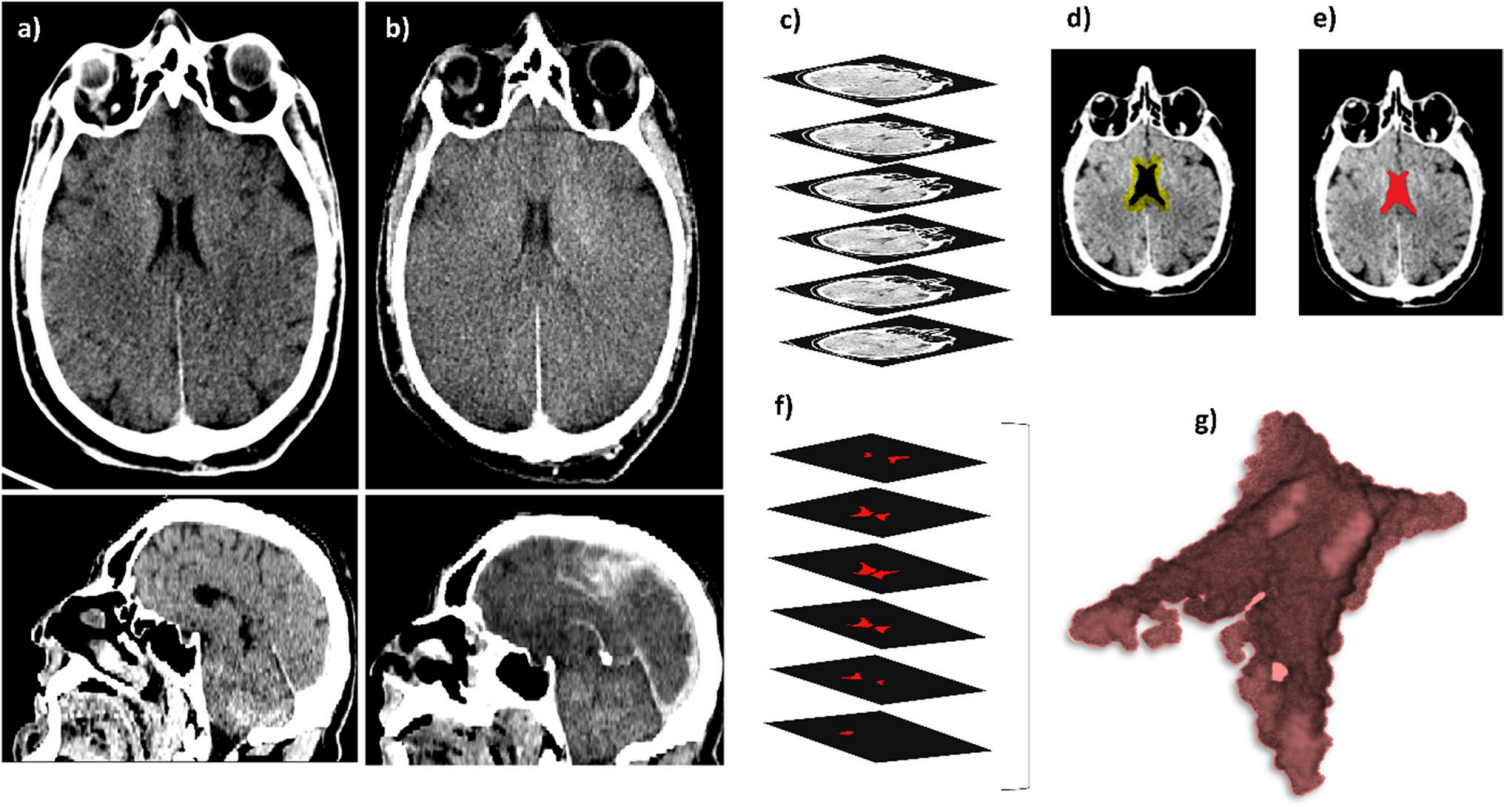
PMCT images in the axial (upper) and in the sagittal (lower) planes of the same representative brain slice at different times after death, namely 24 h (**a**) and 114 h (**b**). In **c**, a graphical representation of the input image set for the segmentation algorithm is shown; for each selected image, a contour (**d**) and a mask (**e**) of the ventricle region for each CT slice are shown. The extrapolated masks of the ventricle slices are represented (**f**), and three-dimensional reconstruction of the cerebral lateral ventricle is shown (**g**)

A total of ten CT image series (one series per subject) were acquired. In Fig. 1, we report two representative axial and sagittal planes obtained on the same subject at different times, namely 24 (a) and 114 (b) h after death. A qualitative analysis of Fig. 1a and b highlights a reduction in the LCV size and a change in its radiodensity. This finding is in close agreement with the results of Hasegawa and co-workers, which previously studied the time-dependent changes in LCV volume and radiodensity on three subjects between 2 and 24 hpm [11]. In this study, the authors found a linear decrease in the LCV volume associated with a linear increase in its radiodensity in the investigated postmortem interval. The results in Fig. 1a and b strongly suggest that the changes reported by Hasegawa and colleagues could continue even after 1 day from the clinical death, thus being of potential help in the estimation of the postmortem interval. However, the analysis of a single CT plane shown in Fig. 1 cannot be considered representative of the whole LCV changes; therefore – following Hasegawa et al. – we carried out a 3D segmentation of the entire LCV as described in material and methods, and we measured its volume and radiodensity over time up to approximately 270 hpm. Figure 1c–g summarize organically the segmentation process adopted to analyze the LCV, acquired from the PMCT images. As fully described in the Material and methods section, a segmentation algorithm implemented in the ImageJ software has been used to label the ventricle region, contouring and masking the related areas per single slice using image thresholding techniques; the steps of this process are depicted in Fig. 1c–e. A representative set of extrapolated masks of the ventricle slices are shown in Fig. 1f, and a representative three-dimensional reconstruction of the cerebral lateral ventricle after death is shown in Fig. 1g. This procedure has been repeated for each time point, for a total of ten available cases.

**Fig. 2:**
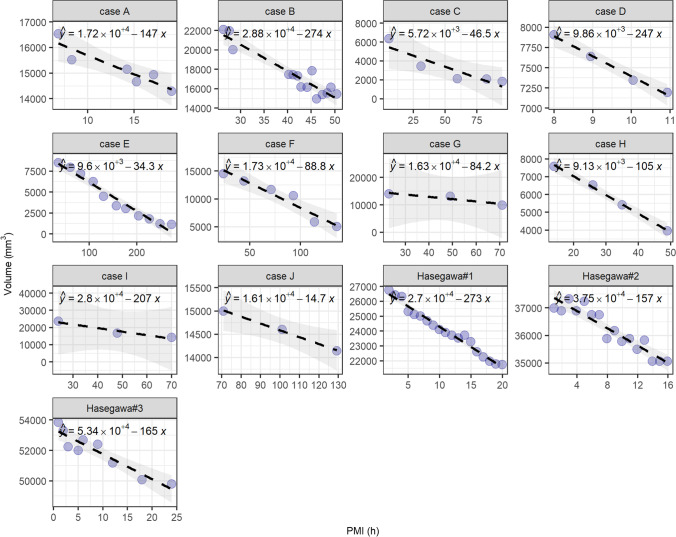
Plots of the LCV volume time evolution for the whole set of analyzed cases compared with the three cases discussed in Hasegawa et al.[11]. A linear fit has been superimposed upon each data set, along with 95% confidence bands

In Fig. 2, we show the time evolution of the LCV volume, expressed in mm^3^. Data lie in the range 5.5–273 hpm. We compared our data with the data of Hasegawa et al., which lie in the range of 2–24 h. Each case is shown separately and identified with a letter from A to J, as far as our measures are concerned. A different notation is used for the cases obtained from the paper of Hasegawa et al., which have been obtained from the published paper as described in the Statistical analysis section.

**Fig. 3:**
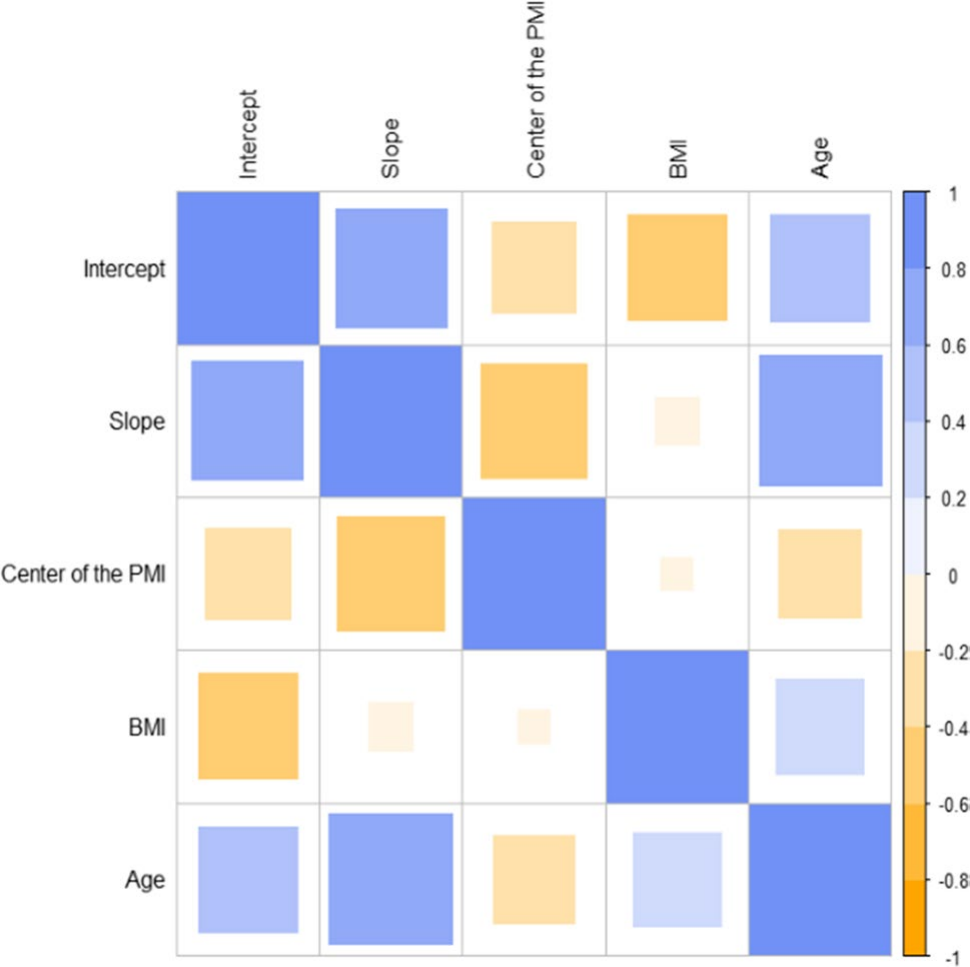
Correlation analysis of the linear regression results (intercept and slope) evaluated for the volume dynamics as well as the baseline parameters of the subjects (age, BMI); the variable “center of the PMI” has been introduced in the analysis to account for an estimate of how the death time relates to the LCV volume evolution

Consistently with Hasegawa and co-workers, we observe a linear decrease in the LCV volume for all the cases. Linear regression analysis is performed on the data, and the results are summarized in Table 2. From the analysis of Table 2 and Fig. 2, one can notice that each subject displays a different linear behavior, with different intercepts and slopes. For the sake of completeness, in Fig. 3, we show a correlation analysis of the linear regression results with the baseline parameters of the subjects (age, height, and weight). Unfortunately, it was not possible to acquire data in the entire investigated range for each subject. To account for the contribution due to such a sampling inhomogeneity, we also included in the correlational analysis the variable “center of the PMI”, which indicates the middle of the investigated PMI range for each corpse. We do not discuss the correlation among the baseline parameters, because it is out of the scope of the present paper. We just notice that the expected correlations are verified. We observe a strong positive correlation between absolute slopes and intercepts, which suggests that larger LCVs decrease faster. We observe a strong negative correlation between the absolute slope and the center of the investigated PMI, indicating that the volume reduction rate slows down over time. This finding requires a more in-depth discussion. The observed negative correlation suggests that the LCV volume evolution is not linear in time. Very likely, we would detect a different behavior if we were able to plot the entire postmortem interval between 0 and 270 hpm. For these time series, an exponential decay could be an educated guess. The fact that we were able to fit properly the data with a linear trend (Fig. 2) indicates that the volume reduction rate changes very slowly over time. This aspect is interesting and deserves a more in-depth study as it might provide valuable information for very large PMI, a time window for which a more quantitative approach is highly required. Looking at the linear trends in Fig. 2, one might be tempted to use the intercept to estimate the LCV volume at the time of the death. Unfortunately, this estimation is not possible because of the decreasing reduction rate over time discussed above, which might lead to a volume underestimation that becomes more relevant moving the PMI forward in time. These hypotheses are further confirmed by the moderate negative correlation between the center of the PMI and intercepts.

**Table 2:** Estimate of the linear coefficients (intercept and slope) of the volume evolution for all cases, as represented in Fig. 2, along with the statistical errors and the *p*-values

		Estimate [mm^3^]	Std. error [mm^3^]	*p* value		Estimate [mm^3^ h^−1^]	Std. error [mm^3^ h^−1^]	*p* value
Case A	(Intercept)	17.2∙10^3^	0.5∙10^3^	2.93E-06	Slope	− 147	32	9.99E-03
Case B	(Intercept)	28.8∙10^3^	1.1∙10^3^	9.70E-12	Slope	− 274	27	3.34E-07
Case C	(Intercept)	5.7∙10^3^	0.8∙10^3^	5.33E-03	Slope	− 47	12	3.24E-02
Case D	(Intercept)	9.86∙10^3^	0.16∙10^3^	2.73E-04	Slope	− 247	17	4.75E-03
Case E	(Intercept)	9.60∙10^3^	0.4∙10^3^	1.37E-09	Slope	− 34	2	9.84E-08
Case F	(Intercept)	17.3∙10^3^	1.1∙10^3^	8.27E-05	Slope	− 89	12	1.71E-03
Case G	(Intercept)	16.3∙10^3^	1.7∙10^3^	6.43E-02	Slope	− 84	32	2.32E-01
Case H	(Intercept)	9.13∙10^3^	0.15∙10^3^	2.73E-04	Slope	− 105	5	1.83E-03
Case I	(Intercept)	28.0∙10^3^	2.5∙10^3^	5.70E-02	Slope	− 207	49	1.49E-01
Case J	(Intercept)	16.1∙10^3^	0.1∙10^3^	3.74E-03	Slope	− 14.7	1.0	3.96E-02
Hasegawa#1	(Intercept)	27.0∙10^3^	0.13∙10^3^	2E-18	Slope	− 273	10	3E-14
Hasegawa#2	(Intercept)	37.5∙10^3^	0.15∙10^3^	2E-16	Slope	− 157	15	7 E-08
Hasegawa#3	(Intercept)	53.4∙10^3^	0.26∙10^3^	1.8E-14	Slope	− 165	23	1.7E-04

**Fig. 4:**
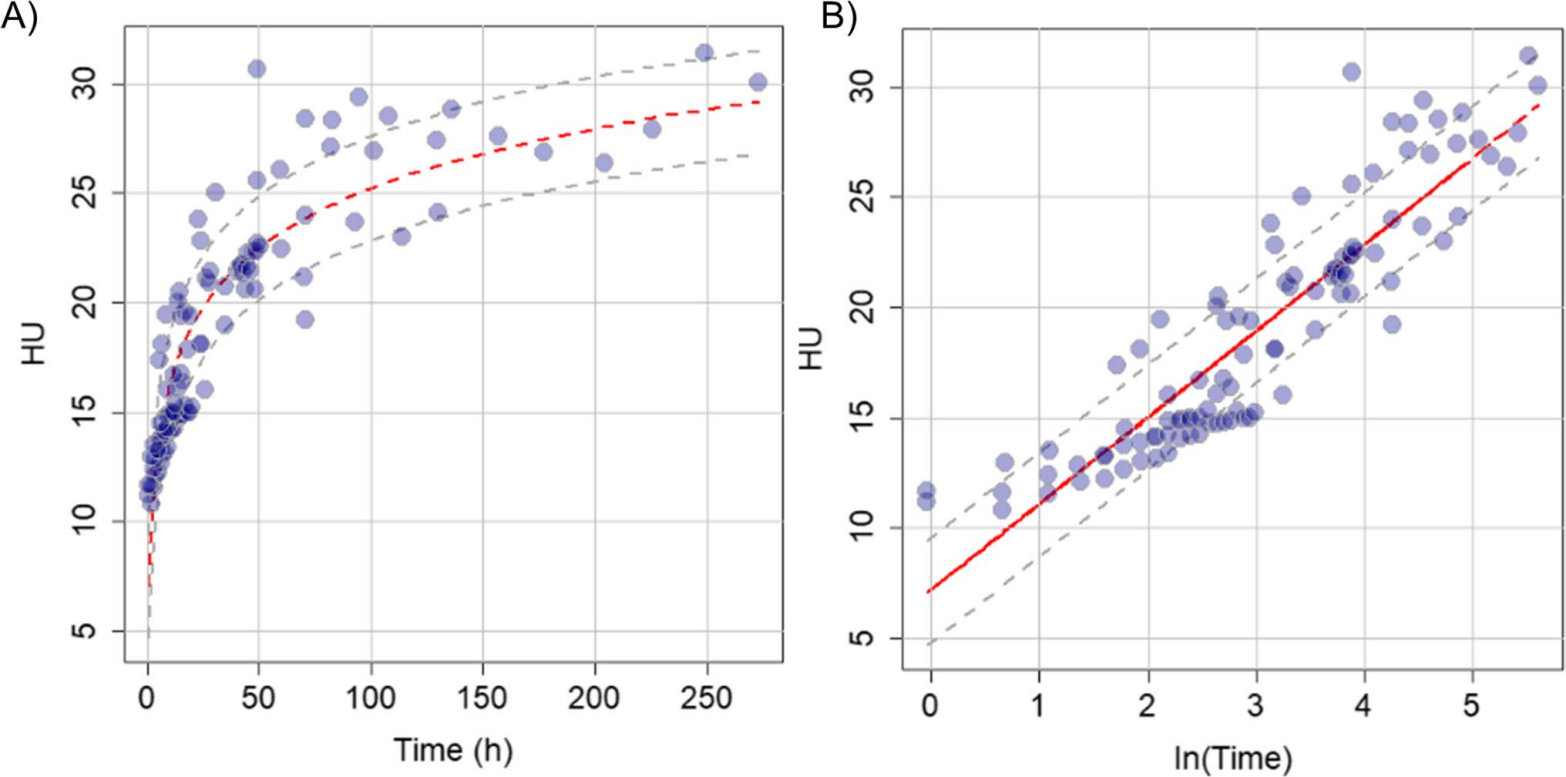
Global plot of the radiodensity evolution for all cases, both in linear (**a**) and in logarithmic (**b**) scales. A logarithmic fit is superimposed to the data together with the corresponding confidence bands. Data measured in this paper are plotted together with data from Hasegawa et al. [11] acquired in the time interval 0–24 hpm. The joint set of data is well fitted by the same logarithmic master curve

In Fig. 4a, we report the time evolution of the average radiodensity measured on the segmented volume. Data are reported in Hounsfield units (HU). All the ten investigated subjects are displayed in the same graph together with the three subjects investigated by Hasegawa and co-workers [11]. Despite the inter-individual variability, the different subjects display a rather consistent time behavior. In particular, one can notice a fast radiodensity increase in the first 48 h after death. Then, we observe a progressive slow down over time, hinting at a saturation phenomenon. To the best of our knowledge, this behavior has never been observed in the literature; therefore, we do not have any further information on possible theoretical models that can be fitted to the data. Moreover, given the substantially scattered nature of the experimental points, especially at large PMI, we tried to describe the data keeping the model as simple as possible. To this purpose, in Fig. 4b, we replicate Fig. 4a using a logarithmic scale on the *x*-axis. In this representation, we observe a roughly linear increase of the data, suggesting that the radiodensity follows a logarithmic increase over time, in the investigated time range. A logarithmic fit is reported in red in both plots together with the corresponding confidence bands. The fit and the confidence bands show a good agreement with the data. Fit parameters are reported in Table 3.

**Table 3:** Estimate of the linear coefficients (intercept and slope) of the radiodensity evolution in logarithmic scale for all cases, as represented in Fig. 4, along with the statistical errors and the *p*-values

	Estimate	Std	t-value	*p*
Intercept	7.20	0.61	11.74	< 2e-16
Slope	3.91	0.19	20.86	< 2e-16
Residual standard error: 2.36 on 101 degrees of freedom Multiple R-squared: 0.811, adjusted R-squared: 0.81				
F-statistic: 435.1 on 1 and 101 DF, *p*-value: < 2.2e-16				

Interestingly the model allows estimating an average radiodensity of 7.1 HU (SD 0.6), which is perfectly consistent with the expected value for a living person.

## Discussion

In this paper, we investigate postmortem changes in the LCV up to approximately 270 hpm. LCV changes were assessed in terms of volume and average radiodensity, as measured through quantitative analysis of PMCT images. It is worth recalling that we had no chance to observe any of the included subjects for the entire time range (0–270 hpm). Conversely, different corpses were observed for different postmortem intervals.

In close agreement with the results of Hasegawa and co-workers [11], we detected a linear decrease in the LCV volume over time for each corpse in the specific investigated postmortem intervals (Fig. 2). A correlational analysis (Fig. 3) shows that absolute slopes retrieved from linear regression changes over time: the larger the center of the investigated PMI, the smaller the absolute slopes. This result hints at a non-linear behavior of the LCV volume over the entire time range (0–270 hpm), which could be of some help in the determination of the time of the death. A more in-depth study is required to verify this hypothesis and to choose the appropriate mathematical framework for analyzing these kinetical values. Unfortunately, the LCV volume is affected by a strong inter-individual variability that is likely to limit its applicability to PMI estimation, as it depends on many factors (Fig. 2) including subject age and sizes. Therefore, we decided to investigate also the change in the LCV average radiodensity of this region. Despite the inter-individual variability, the average LCV radiodensity measured on different subjects collapses onto a single master curve, which is well fitted by a logarithmic trend characterized by an initial fast growth rate, which slows down with time. About this model, a caveat is necessary. We feel that this time trend cannot be used to extrapolate HU values outside the measured interval, because the logarithmic function diverges for infinite times, which is not physically meaningful in this case, while we expect a saturation at approximately 40–50 HU that corresponds to the gray matter. Therefore, we hypothesize that – for larger times – other phenomena might contribute to the HU kinetics, leading to the expected saturation.

Taken all together, the data here presented shows that brain CT has the potential to positively impact the development of novel techniques for PMI determination. In this regard, it seems appropriate to emphasize that the use of CT as a method of studying bodies after death presents both advantages and disadvantages. PMCT is surely less time-consuming compared to an autopsy; it is widely available and non-invasive and necessitates short acquisition times. PMCT data can be stored and revisited indefinitely; furthermore, various modalities can be employed, each able to provide different types of information and insights on ongoing investigations [18]. Overall, PMCT can be extremely useful in many different situations and can surely help when it comes to the determination of the time and cause of death [19, 20]. Concerning the PMI, the latter is typically estimated by exclusion using traditional methods; however, the reliability of these methods is rather poor, and the overall accuracy and precision of the estimation can be enhanced by combining different methods, including postmortem imaging techniques [21]. For instance, compared to rigor and algor mortis which only rarely affect CT findings, livor mortis can be observed and typically manifests as areas of increased attenuation on PMCT. It has also been observed that postmortem structural changes (i.e., corneal thickness) within the eye may help determine the PMI [22].

Nonetheless, postmortem imaging techniques also present some limitations: first of all, the high costs and low availability of CT scanners and personnel. Not all forensic departments have their own CT scanner [23], and there are still no precise guidelines concerning the methods for reporting and addressing forensic radiologic imaging results [24]. Furthermore, being forensic radiology a new discipline, there is still a general lack of experience when it comes to the interpretation of images. Indeed, the evaluation and analysis of PMCT images require a careful consideration of the possible occurring postmortem changes that can occasionally hamper diagnoses or conceal findings. For instance, different body components may display similar HU values (i.e., gray matter, 40–50 HU; blood, 40–80 HU [25]), and this could alter results. Moreover, imaging procedures may sometimes be bound to specific study conditions (i.e., room temperature and humidity, body position) and this could be a problem in terms of reproducibility of results. In this context, we feel that a multiparametric and multiscale approach, such as the one employed in the Swiss project “Virtopsy” that combined different radiological techniques with finding from forensic medicine, pathology, physics, and biomechanics [26], would help to provide new insights into the complex problem of determining the postmortem interval.

## Conclusion

Here, we investigate postmortem changes in the lateral cerebral ventricle through the quantitative analysis of PMCT images. Our results show a decrease over time in the volume of the LCV after death, in agreement with previous studies [11]. Additionally, larger LCVs seem to decrease faster than smaller ones, and the reduction of the LCVs gets slower over time. Overall, the volume kinetics appears to be affected by a large inter-individual variability depending on many factors, including age and subject size. The results also point out a temporal increase of CSF radiodensity after death. Interestingly, data acquired on different subjects appear to collapse into a single time-dependent master curve, which is well fitted by a logarithmic increase, albeit with large 95% confidence bands. The reliability of this mathematical description is confirmed by the estimated radiodensity at the time of the death, which is consistent with the expected values in living subjects. These results suggest that the study of the average radiodensity evolution over time could be more effective than the study of the LCV volume for determining the PMI.

Taken altogether, our results show that the analysis of the postmortem variation of CSF radiodensity can be a potentially useful tool in determining postmortem interval.
